# Designing Efficient Spaced Seeds for SOLiD Read Mapping

**DOI:** 10.1155/2010/708501

**Published:** 2010-09-16

**Authors:** Laurent Noé, Marta Gîrdea, Gregory Kucherov

**Affiliations:** ^1^INRIA Lille-Nord Europe, LIFL/CNRS, Université Lille 1, 59655 Villeneuve d'Ascq, France; ^2^J.-V.Poncelet Laboratory, Bolshoy Vlasyevsky 11, Moscow 119002, Russia

## Abstract

The advent of high-throughput sequencing technologies constituted a major advance in genomic studies, offering new prospects in a wide range of applications.We propose a rigorous and flexible algorithmic solution to mapping SOLiD color-space reads to a reference genome. The solution relies on an advanced method of seed design that uses a faithful probabilistic model of read matches and, on the other hand, a novel seeding principle especially adapted to read mapping. Our method can handle both lossy and lossless frameworks and is able to distinguish, at the level of seed design, between SNPs and reading errors. We illustrate our approach by several seed designs and demonstrate their efficiency.

## 1. Introduction

High-throughput sequencing technologies can produce hundreds of millions of DNA sequence reads in a single run, providing faster and less expensive solutions to a wide range of genomic problems. Among them, the popular SOLiD system (Applied Biosystems, Life Technologies) features a 2-base encoding of reads, with an error-correcting capability helping to reduce the error rate and to better distinguish between sequencing errors and SNPs.

In this paper, we propose a rigorous and flexible algorithmic approach to mapping SOLiD color-space reads to a reference genome, capable to take into account various external parameters as well as intrinsic properties of reads resulting from the SOLiD technology. The flexibility and power of our approach comes from an advanced use of *spaced seeds* [1, 2].

The main novelty of our method is an *advanced seed design* based on a *faithful probabilistic model of SOLiD read alignments* incorporating reading errors, SNPs, and base indels, and, on the other hand, on a *new seeding principle* especially adapted for read mapping. The latter relies on the use of a small number of seeds *designed simultaneously with a set of positions on the read where they can hit*. We call this principle *position-restricted seeds*. Advantageously, it allows us to take into account, in a subtle way, read properties such as a nonuniform distribution of reading errors along the read, or a tendency of reading errors to occur periodically at a distance of 5 positions, which are observed artifacts of the SOLiD technology.

A number of algorithms and associated software programs for read mapping have been recently published. Several of them such as MAQ [3], MOSAIK [4], MPSCAN [5] PASS [6], PerM [7], RazerS [8], SHRiMP [9], or ZOOM [10] apply contiguous or spaced seeding techniques, requiring one or several hits per read. Other programs approach the problem differently, for example, by using the Burrows-Wheeler transform (Bowtie [11], BWA [12], SOAP2 [13]), suffix arrays (segemehl [14], BFAST [15]), variations of the Rabin-Karp algorithm (SOCS [16]) or a nondeterministic automata matching algorithm on a keyword tree of the search strings (PatMaN [17]). Some tools, such as segemehl [14] or Eland [18], are designed for 454 and Illumina reads and thus do not deal with the characteristics of the SOLiD encoding which is the subject of this paper. Also, it should be noted that, in many cases, sensitivity is sacrificed in favor of speed: most methods find similarities up to a small number of mismatches, and few approaches account for nucleotide insertions and deletions.

Seed-based methods for read mapping use different seeding strategies. SHRiMP [9] uses spaced seeds that can hit at any position of the read and introduces a lower bound on the number of hits within one read. MAQ [3] uses six light-weight seeds allowed to hit in the initial part of the read. ZOOM [10] proposes to use a small number (4–6) of spaced seeds each applying at a fixed position, to ensure a lossless search with respect to a given number of mismatches. In the lossless framework, PerM [7] proposes to use “periodic seeds” (see also [19]) to save on the index size.

Despite the number of proposed solutions, none of them relies on a systematic seed design method taking into account (other than very empirically) statistical properties of reads. In this paper, we present a seed design based on Hidden Markov models of read matches, using a formal finite automata-based approach previously developed in [20]. To the best of our knowledge, this is the first time that the seed design for read mapping is done based on a rigorous probabilistic modeling.

Our approach allows us to design seeds in both lossy and lossless frameworks. In the lossless framework, where the goal is to detect all read occurrences within a specified number of mismatches, we have the flexibility of partitioning this number into reading errors and SNPs.

As a result, we obtain a very efficient mapping algorithm combining a small number of seeds and therefore a reasonable amount of index memory with guaranteed sensitivity and small running time, due to a restricted subset of positions where seeds should be applied.

## 2. AB SOLiD Reads: Encoding and Technological Artifacts

The SOLiD System [21] enables massively parallel sequencing of clonally amplified DNA fragments linked to beads. This sequencing technology is based on sequential ligation of dye-labeled oligonucleotide probes, each probe determining two base positions at a time. The system uses four fluorescent dyes to encode for the sixteen possible 2-base combinations. Consequently, a DNA fragment is represented by the initial base followed by a sequence of overlapping dimers, each encoded with one of four colors using a degenerate coding scheme that satisfies several rules. Thus, although a single color in a read can represent any of four dimers, the overlapping properties of the dimers and the nature of the color code eliminate ambiguities and allow for error-correcting properties.

As our work relies on modeling the error distribution along the reads, we are particularly interested in several aspects of the sequencing technology that influence this distribution.

First, since every color of the read encodes two adjacent bases and therefore every base affects two adjacent colors, it follows that any single base mutation results in the change of two adjacent colors in the read.

Second, regarding reading errors, the sequencing chemistry (described in [21, 22]) suggests a periodical bias along the read. Basically, the sequencing by ligation process within the SOLiD platform relies on successive hybridizations of 8-mer oligonucleotides on the template to be sequenced. The oligonucleotides contain 3 universal base, 3 degenerate bases and 2 adjacent bases that identify two positions on the template, correlated with the identity of the fluorescent labels at their 5′ end. After ligation, bases 6–8 are cleaved off, along with the fluorescent dye, leaving the 5′ end available for another ligation. Hence, two positions *p* and *p* + 1 are correctly base paired after attaching one oligonucleotide, and the positions at distance 5 (*p* + 5 and *p* + 6) are determined by the next oligonucleotide. The nucleotides at positions that do not fit this pattern are determined in subsequent rounds. Five rounds consisting of several ligation cycles are necessary to cover the template. Therefore, we expect reading error biases to propagate during such a sequencing round, thus appearing with a periodicity of 5.

To confirm this intuition, we studied the variation of the reading error probability along the read by analyzing statistical properties of about a million of SOLiD reads of the *Saccharomyces cerevisiae* genome. In this analysis, we used the qualities *Q*_*l*_ associated to each position *l* on the read, which relate to the error probability *p*_*e*_^*l*^ through *Q*_*l*_ = −10 · log _10_(*p*_*e*_^*l*^) [23].

**Figure 1:**
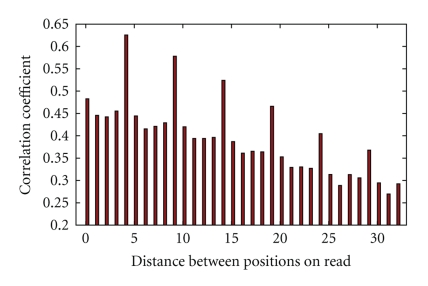
Position quality correlation coefficient depending on the distance between read positions.

 We computed the quality correlation between read positions depending on the distance between them. Formally, if *m* is the read length, then for each *i* ∈ {1,…, *m* − 1}, we computed the correlation through the following standard formula $c(i)=E((Q_{j}-\tilde{Q})(Q_{j+i}-\tilde{Q}))/{\left(\sigma_{Q}\right)}^{2}$, where *E*(·) is the expectation, $\tilde{Q}$ the average quality along the read, and *σ*_*Q*_ the standard deviation of quality values. The result is given in Figure 1. It shows significantly higher correlations (up to 0.63) between pairs of positions located at distances that are multiples of 5.

**Figure 2:**
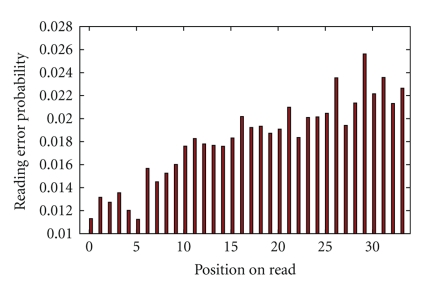
Average reading error probability at each read position.

 Additionally, we studied the behavior of reading error probability values along the read. As shown in Figure 2, the error probability tends to increase towards the end of the read, making the last positions of the color sequence less reliable when searching for similarities.

## 3. Seed Design for Mapping SOLiD Reads

### 3.1. Seed Design: Background

Spaced seeds, first proposed in the context of DNA sequence alignment by the PatternHunter algorithm [1], represent a powerful tool for enhancing the efficiency of the sequence search.

Using a spaced seed instead of a contiguous stretch of identical nucleotides to select a potential similarity region can improve the sensitivity of the search for a given selectivity level [1]. Furthermore, using a seed family, that is, several seeds simultaneously instead of a single seed, further improves the sensibility/selectivity tradeoff [24, 25]. The price for using seed families is the necessity to store in memory several indexes, one for each seed. In practice, however, using in the search a small number of seeds can significantly improve the sensitivity/selectivity ratio.

A crucial feature of spaced seeds is their capacity to be adapted to different search situations. Spaced seeds can be *designed* to capture statistical properties of sequences to be searched. For example, [26, 27] report on designing spaced seeds adapted to the search of coding regions. One of the contributions of this paper is a rigorous design of seeds adapted to mapping genomic reads issued from the SOLiD technology. Note that here we will work with regular spaced seeds rather than more advanced subset seeds [20, 27, 28], as there is very little or no information in discriminating among different classes of mismatches that can be used to our advantage.

One has to distinguish between the *lossy* and *lossless* cases of seed-based search. In the lossy case we are allowed to miss a fraction of target matches, and the usual goal of seed design is to maximize the sensitivity over a class of seeds verifying a certain selectivity level. In the lossless case we must detect all matches within a given dissimilarity threshold (expressed in terms of a number of errors or a minimal score), and the goal of seed design is to compute a minimal set of seeds with the best selectivity that still ensures the lossless search. In the context of read mapping for High-throughput sequencing technologies, both lossy [3, 9] and lossless [7, 10] frameworks have been used.

Our approach to seed design relies on a methodology proposed in our previous work [20], based on the finite automata theory. A central idea is to model the set of target alignments by a  *finite-state* *probability transducer*, which subsumes the Hidden Markov Model commonly used in biosequence analysis. On the other hand, a seed, or a seed family, is modeled by a *seed automaton* for which we proposed an efficient compact construction [29]. Once these two automata have been specified, computing the seed sensitivity can be done efficiently with a dynamic programming algorithm as described in [20]. The seed design is then done by applying our Iedera software [20, 29, 30] that uses the above algorithm to explore the space of possible seeds and select the most sensitive seeds using a sampling procedure for seeds and respective hit positions and by performing a local optimization on the best candidates.

Here we apply this methodology to seed design for mapping SOLiD reads, both in the lossy and lossless frameworks. Besides, we introduce an important novelty in the definition of seeds, especially advantageous for mapping short reads: *position-restricted seeds*, which are seeds designed together with the set of positions on the read where they can be applied. This can be seen as an intermediate paradigm between applying each seed at every position and the framework of [10] where each seed applies to a designated position of the read. Position-restricted seeds offer an additional power of capturing certain read properties (such as, an increasing error level towards the end of the read) in a flexible way, without sacrificing the selectivity and thus the speed of the seeding procedure. A preliminary version of this work is described in [31].

### 3.2. Modeling Seeds and SOLiD Reads by Finite Automata

We now present our model of color sequence alignments, built on the observations of Section 2. Note that we consider the reference genome translated into the color alphabet, that is, both the reads and the genome are represented in color space.

#### 3.2.1. Position-Restricted Seeds

As shown in Section 2, the reading error probability increases towards the end of the read, implying that a search for similarity within the last positions of the read could lead to erroneous results or no results at all. Hence, we can improve the seed selectivity by favoring hits at initial positions of the read where matches are more likely to be significant. We then define each seed *π* *jointly* with a set of positions *P* to which it is applied on the read.

We use the framework of [20] where a seed *π* is represented by a deterministic finite automaton 𝒬 over the alignment alphabet *𝒜* which is here the binary match/mismatch alphabet. Note that the size of 𝒬 is a crucial parameter in the algorithm of [20] for computing the sensitivity of the seed. An efficient construction of such an automaton has been studied in [29]: it has the optimal size of (*w* + 1)2^*s*−*w*^ states, where *s* and *w* are, respectively, the *span* (length) and *weight* (number of *match* symbols) of the seed.

Let *m* be the read size. To take into account the set of allowed positions, we compute the product of 𝒬 with an automaton *λ*_*P*_ consisting of a linear chain of *m* + 1 states *q*_0_, *q*_1_,…, *q*_*m*_, where *q*_0_ is the initial state, and for every *q*_*i*_, both outgoing transitions lead to *q*_*i*+1_. Final states of the automaton reflect the set of possible positions *P* where the seed is allowed to hit: a state *q*_*i*_ is final iff *i* − *s* ∈ *P*.

A trivial upper bound on the size of the product automaton for a spaced seed of span *s* and weight *w* is (*w* + 1) · 2^*s*−*w*^ · *m*. This bound can be improved using the notion of matching prefix, as explained in [29]. Thus, an economical implementation of the product of 𝒬 by *λ* taking into account the set of matching positions *P* always produces at most ((*w* + 1) · 2^*s*−*w*^·|*P* | + *m*) states.

 Furthermore, consider an interval graph of the possible placements of the seed on the read, where each placement spans over an interval of *s* positions. The chromatic number *c* of this graph can be easily computed, providing the maximal number of overlapping seeds. We observe that if this number is small (compared to (*s* − *w* + log (*w*))), then the size of the product automaton is bounded by *O*((*m* + 1) · 2^*c*^).

#### 3.2.2. Model for SNPs and Reading Errors

As explained in Section 2, there are two independent sources of errors in reads with respect to the reference genome: reading errors and SNPs/indels, that is, *bona fide* differences between the reference genome and sequenced data. We represent each of these sources by a separate Hidden Markov Model (viewed as a probabilistic transducer, see [20]), combined in a model which allows all error types to be cumulated in the resulting sequences.

**Figure 3:**
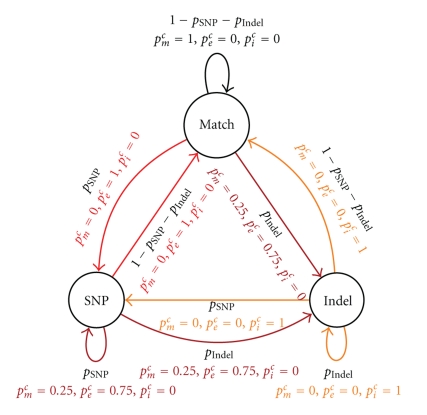
Model of SNPs and Indels (*M*_SNP/*I*_). Colors of transitions correspond to emitted errors: black for color matches, red for mismatches, yellow for indels, and dark red for a mixture of matches (0.25) and mismatches (0.75).

The **SNP/Indel model**, denoted *M*_SNP/*I*_, (Figure 3) has three states: *Match*, *SNP* and *Indel*, referring to matches, mismatches, and indels *at the nucleotide level*, and is parameterized by SNP and Indel occurrence probabilities, denoted *p*_SNP_ and *p*_Indel_. Each transition of *M*_SNP/*I*_ generates a *color match, mismatch or indel*, with probabilities *p*_*m*_^*c*^, *p*_*e*_^*c*^, and *p*_*i*_^*c*^, respectively, defined as follows. An insertion or deletion of *n* nucleotides appears at the color level as an insertion/deletion of *n* colors preceded in 3/4 cases by a color mismatch [21]. Hence, the *p*_*e*_^*c*^ = 0.75 for transitions incoming to the *Indel* state, and *p*_*i*_^*c*^ = 1 for any transition outgoing from the *Indel* state. A nucleotide mutation is reflected in the color encoding by a change of two adjacent colors (and, more generally, *n* consecutive mutations affect *n* + 1 consecutive colors [21]). Thus, *p*_*e*_^*c*^ = 1 when entering or leaving the *SNP* state, and a color match/mismatch mixture when staying in the mismatch state, since color matches may occur inside stretches of consecutive SNPs. Finally, *p*_*m*_^*c*^ = 1 when looping on the *M* state.

**Figure 4:**
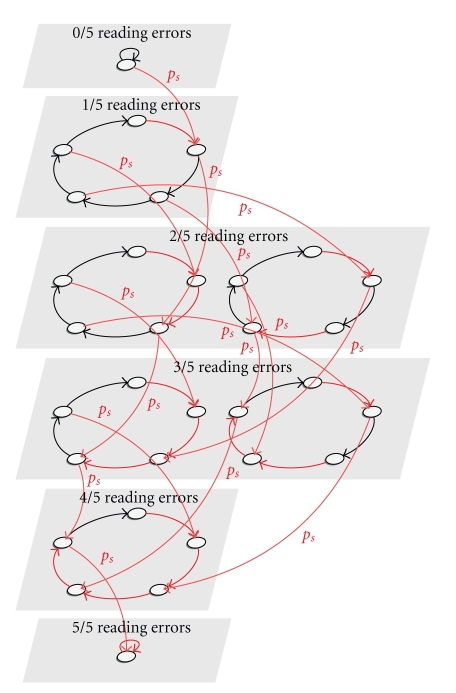
Reading error automaton.

The **reading errors** are handled by a more complex model, denoted *M*_RE_ (Figure 4). Basically, it is composed of several submodels, one for each possible arrangement of reading errors on a cycle of 5 positions. Within these submodels, the transitions shown in red correspond to periodic reading errors, and generate reading errors with a fixed probability *p*_err_. This simulates the periodicity property shown in Figure 1. Switching from one cyclic submodel to another with a higher reading error rate (by adding another red transition with high error probability) can occur at any moment with a fixed probability *p*_*s*_.

 The transitions shown in black in the model in Figure 4 have an error emission probability of 0. However, in the complete reading error model, we wish to simulate the error probability that increases towards the end (in conformity with Figure 2). We do this by ensuring that reading errors are generated by these transitions with a probability *p*_err_′(pos) (lower than *p*_err_) given by an increasing function of the current position *p* *o* *s* on the read. Technically, this is achieved by multiplying the automaton in Figure 4 by a linear automaton with *m* + 1 states, where *m* is the read length and the *i*th transition generates a reading error (color mismatch) with the probability *p*_err_′(*i*). The reading error emission probability in the product model is computed as the maximum of the two reading error probabilities encountered in the multiplied models.

The **final model**, which combines both error sources, is the product of *M*_SNP/*I*_ and *M*_RE_. While the states and transitions of the product model are defined in the classic manner, the emissions are defined through specific rules based on symbol priorities. If corresponding transitions of *M*_SNP/*I*_ and *M*_RE_ generate symbols *α* and *β* with probabilities *p*_1_ and *p*_2_, respectively, then the product automaton generates the dominant symbol between *α* and *β* with probability *p*_1_ *p*_2_. Different probabilities obtained in this way for the same symbol are added up.

The dominance relation is defined as follows: *indels* are dominant over both *mismatches* and *matches*, and *mismatches* dominate *matches*. For example, (*i* *n* *d* *e* *l*, *m* *i* *s* *m* *a* *t* *c* *h*) results in an *i* *n* *d* *e* *l*, (*m* *i* *s* *m* *a* *t* *c* *h*, *m* *i* *s* *m* *a* *t* *c* *h*) and (*m* *a* *t* *c* *h*, *m* *i* *s* *m* *a* *t* *c* *h*) represent *m* *i* *s* *m* *a* *t* *c* *h*, (*m* *a* *t* *c* *h*, *m* *a* *t* *c* *h*) is a *m* *a* *t* *c* *h*. This approach ensures that errors generated by each of the two models are superposed.

### 3.3. Computing the Sensitivity and Testing the Lossless Property

Given an automaton 𝒬 specifying a family of seeds possibly restricted to a set of positions, we have to compute its sensitivity (in the lossy framework) or to test whether it is lossless (in the lossless framework).

#### 3.3.1. Sensitivity

The sensitivity of a seed family is defined [1, 32] as the probability for at least one of the seeds to hit a read alignment with respect to a given probabilistic model of the alignment. As outlined in Section 3.1, this is done using the dynamic programming technique of [20]. We therefore omit further details.

#### 3.3.2. Efficient Algorithm for Testing the Lossless Property

In the lossless framework, we have to test if the seed specified by 𝒬 is lossless, that is, hits *all* the target alignments. The set of target alignments is defined through a threshold number of allowed mismatches.

A straightforward way to test the lossless property of 𝒬 would be to construct a deterministic automaton recognizing the set of all target alignments and then to test if the language of this automaton is included in the language of 𝒬. This, however, is unfeasible in practice. The automaton of all target alignments is much too costly to construct: for example, in the case of threshold of *k* mismatches, there are $\sum_{a=0}^{k}\left(\begin{array}{c} m\\ a \end{array}\right)$ different alignments of length *m*, and the Aho-Corasick automaton of these strings would have $\sum_{a=0}^{k+1}\left(\begin{array}{c} m\\ a \end{array}\right)$ states. Moreover, testing the inclusion would lead to computing the product of this automaton with 𝒬, which would multiply the number of states of this automaton by the number of states of 𝒬.

Alternatively, we propose an efficient dynamic programming algorithm directly applied to 𝒬 that can verify the inclusion (Algorithm 1). This algorithm computes, for each state *q* of 𝒬, and for each iteration *i* ∈ [1 ⋯ *m*], the minimal number of mismatches needed to reach *q* at step *i*. Let *k* be the threshold for the number of mismatches. Then, the lossless condition holds if and only if, at step *m*, all nonfinal states have a number of mismatches greater than *k*. Indeed, if there is a nonfinal state that has a number of errors at most *k* after *m* steps, then there is at least one string of length *m* with at most *k* mismatches that is not detected by the automaton, which contradicts the lossless condition. This algorithm is of time complexity *𝒪*(|𝒬 | ·|*𝒜* | · *m*), and space complexity *𝒪*(|𝒬 | ·|*𝒜*|), where *𝒜* is the alphabet of the alignment sequences.

To illustrate the efficiency of this algorithm, consider the case of a single spaced seed of span *s* and weight *w*, yielding an automaton with at most (*w* + 1) · 2^*s*−*w*^ states [20, 33]. On this automaton, our method runs in time *O*(*w* *m*2^*s*−*w*^) which brings an improvement by a factor of 2^*w*^/*w* of the general bound *𝒪*(*m*2^*s*^) from [34].

#### 3.3.3. Lossless Seeds with Respect to SNPs and Reading Errors

**Figure 5:**
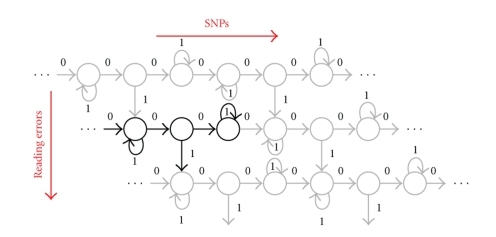
Building an automaton for *k* SNPs and *h* color mismatches from a repeated 3-state pattern.

**Figure 6:**
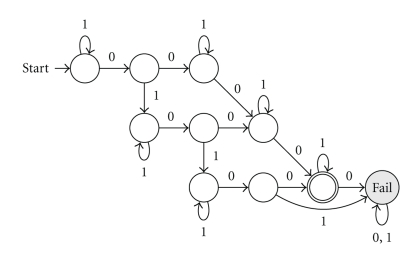
1 SNP and 2 errors automaton.

In the context of color sequence mapping, it is interesting to define the lossless property with respect to a *maximal number of allowed mismatches that is split between SNPs and reading errors*. Since, in the color space, a SNP appears as two adjacent color mismatches, having *k* nonconsecutive SNPs and *h* color mismatches implies the possibility to accept 2*k* + *h* mismatches with the additional restriction that there exist at least *k* pairs of adjacent ones. The automaton that recognizes the set of alignments verifying this condition on mismatches can be obtained by combining simple 3-state building blocks as depicted in Figure 5. An example of such an automaton, accepting 1 SNP and 2 reading errors, is illustrated in Figure 6 (1 and 0 denote *m* *a* *t* *c* *h* and *m* *i* *s* *m* *a* *t* *c* *h* resp.).

Note that the case of consecutive SNPs, resulting in sequences of adjacent color mismatches, is a simpler problem (since consecutive SNPs produce less mismatches in the color representation than the same number of nonconsecutive SNPs) and is covered by the proposed model: a seed that is lossless for alignments with nonconsecutive SNPs will also be lossless for alignments with the same number of consecutive SNPs.

To verify the lossless property for *k* SNPs and *h* color mismatches, we intersect the corresponding automaton with the seed automaton (thus restricting the set of alignments recognized by the seed to those with *k* SNPs and *h* color mismatches) and submit the result to the dynamic programming algorithm described above.

#### 3.3.4. Lossless Seeds with Respect to SNPs, Reading Errors and Indels

In a similar approach, a more complex automaton that takes into account insertions and deletions can be constructed, and intersected with the seed automaton in order to compute its lossless property using the given dynamic programming algorithm. We chose a cost for each event (*m* *a* *t* *c* *h*, *m* *i* *s* *m* *a* *t* *c* *h*—reading error or SNP, and *i* *n* *d* *e* *l*), and with the same reasoning as above we consider alignments under a certain total cost rather than under a certain number of mismatches. In our experiments, we have chosen to use the costs: 0 for match, 2 for one color mismatch, 3 for one SNP (equivalent to having a 1 cost for a color mismatch preceded by another color mismatch), and 4 for indels.

**Figure 7:**
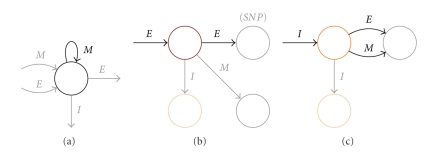
Modeling matches (a), reading errors, SNPs (b), and indels (c). If we consider the costs 0, 2, 3, and 4, respectively: (a) the cost of a match is 0; (b) the cost of single mismatch is 2, and a mismatch that follows it has the cost 1, summing to 3 which is the cost of a SNP; (c) the cost of an indel is 4, and a subsequent mismatch is accepted at zero additional cost.

In Section 3.2, we explained how modifications at the DNA sequence level are visible as differences between the associated color sequences. Following the same principle, we can build automata that represent alignments with a limited number of errors, in various combinations. The building blocks of such automata are represented in Figure 7. Sequences of matches are represented by looping into the same state without any cost modification: (a) SNPs are represented by two consecutive color mismatches with different costs, while reading errors are represented as isolated mismatches, (b) finally, indels of bases correspond to indels of colors that may or may not be followed by a color mismatch, (c) hence color mismatches preceded by an indel event are considered to have zero costs. A complete automaton example is not given here for complexity reasons. The next section will present seeds that are lossless for alignments with cost 7 under the cost associations given above, which accounts for several possible error combinations: 1 *i* *n* *d* *e* *l* and 1 SNP, 1 *i* *n* *d* *e* *l* and 1 *reading error*, 1 SNP and 2 *reading errors*, or 2 SNPs.

Note that our seeds are *not* indel seeds [35], that is, the seed alphabet does not contain a symbol for insertions and deletions. Instead, the seeds must be placed *between* the indels within the alignments modeled by this automaton.

### 3.4. Designed Seeds

We present now several efficient seed designs illustrating our methodology (more examples can be found at http://bioinfo.lifl.fr/yass/iedera_solid).

#### 3.4.1. Lossy Seeds

**Figure 8:**
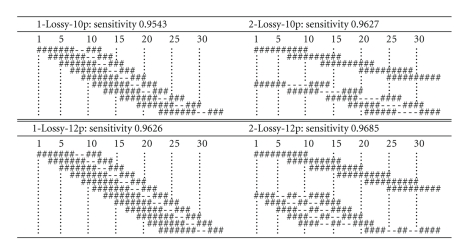
Position-restricted seeds for 10 (above) and 12 (below) allowed positions. Different placements of a seed correspond to the allowed positions.

We first computed several sets of lossy seeds of weight 10, restricted to either 10 or 12 positions among the 34 positions of SOLiD reads, each including one or two seeds. Figure 8 shows some of the resulting seeds, together with the corresponding sensitivity values, computed through the methods described in Section 3.

 Interestingly, both single seeds 1-Lossy-10p and 1-Lossy-12p contain a double gap, which may reflect that an SNP modifies two adjacent colors. However, this gap is not centered but rather shifted at the twothird of the seed (as observed for the best single seeds of [19]). Note also that in the two-seed families 2-Lossy-10p and 2-Lossy-12p, one of the chosen seeds is ungapped. This may be a consequence of the fact that we consider indels in our lossy model, which usually forces the seeds to have a smaller span. Another interesting observation is that two-seed families 2-Lossy-10p and 2-Lossy-12p are actually lossless for the threshold of 3 mismatches, whereas single seeds 1-Lossy-10p and 1-Lossy-12p are not lossless for this setting.

#### 3.4.2. Lossless Seeds for SNPs and Reading Errors

**Figure 9:**
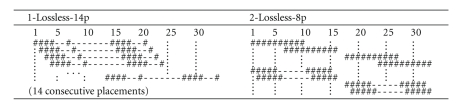
Lossless position-restricted seeds for 1 SNP and 2 reading errors.

We then focused on the lossless case where the maximal number of allowed mismatches is split between SNPs and reading errors. Using the procedure described in Section 3.3, we computed lossless single and double seeds for one SNP and two reading errors. Results are shown in Figure 9. 

Note that the seed 1-Lossless-14p is one of several single seeds of weight 10 we found that satisfied this lossless condition, with no restriction on allowed positions. Interestingly, they all have a very large span (21) and a regular pattern with a periodic structure that can be obtained by iterating a simpler pattern solving the lossless problem for an appropriate *cyclic problem*, following the property we previously described in [19]. For two-seed families, Figure 9 shows a lossless pair of seeds 2-Lossless-8p for read length 33 (which then remains lossless for larger lengths), where each seed is restricted to apply to four positions only.

#### 3.4.3. Lossless Seeds for SNPs, Reading Errors and Indels

**Figure 10:**
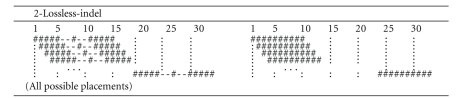
A family of two spaced seeds, lossless for 4 different error combinations: 1 indel and 1 SNP, 1 indel and 1 reading error, and 2 SNPs, or 1 SNP and 2 reading errors.

Figure 10 displays a seed family that is lossless for 1 indel and 1 SNP or 1 indel and 1 reading error, 2 SNPs, or 1 SNP and 2 reading errors. As in the case of lossy seeds where indels are taken into account, these lossless seeds tend to have smaller lengths and therefore fewer gaps, in order to avoid any possible indel position.

#### 3.4.4. Seed Comparison

**Figure 11:**
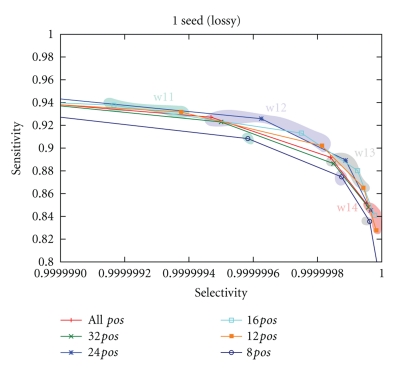
Theoretical selectivity/sensitivity of single seeds. *X*-axis corresponds to selectivity (1 minus the probability of hitting a random alignment) and *Y*-axis to sensitivity. Color clouds highlight seeds of the same weight.

Figure 11 compares the theoretical selectivity/sensitivity of several single seeds, for weight ranging from 11 to 14, depending on the number of read positions the seed can be applied at. Note that restricting the number of possible hitting positions affects the seed template. The red polyline connects points corresponding to seeds optimized without restrictions on the number of positions. Relative to this line, we observe a good performance of seeds restricted to 24, 16 and 12 positions, with corresponding polylines lying above the red one, which means a better sensitivity/selectivity tradeoff. This confirms that position-restricted seeds can be superior to unrestricted seeds, taking advantage of preferentially hitting positions where errors are less likely.

**Figure 12:**
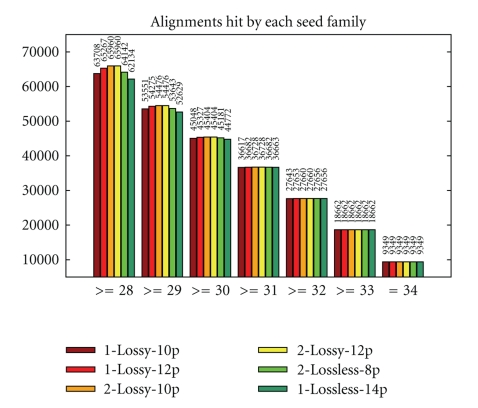
Number of read alignments with scores between 28 and 34 hit by each seed.

Furthermore, to get a better idea of the sensitivity of the obtained seeds applied to real data, we tested them on 100000 reads of length 34 from *Saccharomyces cerevisiae* and computed the number of read alignments hit by each (single or double) seed. Alignments were defined through the score varying from 28 to 34, under the scoring scheme +1 for match, 0 for color mismatch or SNP, -2 for gaps. Results are presented in Figure 12. One conclusion we can draw is that the performance of lossless seeds 1-Lossless-14p and 2-Lossless-8p decreases quite fast when the alignment score goes down, compared to lossy seeds. Intuitively, this is, in a sense, a price to pay for the lossless condition which usually makes these seeds less appropriate for the alignments with a number of errors exceeding the threshold. Another observation is that, as expected, single seeds perform worse than double seeds, although the overall number of positions where seeds apply is the same for both single and double seeds.

Note finally that the choice of the best seed can be affected, on the one hand, by different properties of the class of target alignments (number, type and distribution of mismatches and indels etc.) and, on the other hand, by the size of the data and available computational resources. The former can be captured by our probabilistic models described in Section 3. The latter is related to the choice of the selectivity level, directly affecting the speed of the search, which is defined by the seed weight and the number of allowed positions. Depending on the chosen selectivity, different seeds can (and should) be preferred. Note in this regard that seeds appearing in Figure 12 have different selectivity and are then incomparable *stricto sensu*.

## 4. Experiments

### 4.1. Implementation

Firstly, we briefly present a read mapping tool (SToRM) [36] that we implemented and used for the experiments described below in Section 4.2. A detailed description of the software will be the subject of an accompanying paper.

In our implementation, the data processing is entirely performed in the color space. The reference genome is translated into colors and indexed accordingly. There are four main processing steps.

**Step 1 (seed filtration)**

For each read, candidate positions are identified using seeds designed as explained in Section 3 and corresponding keywords are extracted and looked up in the hash table for the reference genome. Currently, the single-hit strategy is used, that is, one seed hit is sufficient to trigger the further processing.

**Step 2 (SIMD filtration)**

A fast SIMD bandwidth alignment algorithm, that can process several hits in a single run, detects and eliminates spurious hits by discarding candidate mapping positions where the corresponding reference fragment does not show sufficient similarity with the read. This filter uses the sse2 instruction set and implements the local alignment algorithm of Gotoh [37] on *compressed* data (2 bits per color). For example, on reads of length 64 aligned with at most 7 indels (16 diagonals), the filter processes 2 millions hits in less than 1 second, using only one core of a 2.57 GHz Core2 Dual processor.

**Step 3 (alignment)**

The goal of this step is to obtain alignments of color sequences that are meaningful at the nucleotide level, that is, to make the distinction between mismatches caused by reading errors and by SNPs and indels, and to properly assign the corresponding score penalties. This is done using a base-intelligent alignment algorithm which performs a gapped bandwidth alignment between each pair (read, reference sequencing fragment) that passed the filter from *Step 2*. The algorithm is based on the classic semi-global sequence alignment approach, enriched by a *limited memory of the previous color mismatches* on each path of the alignment matrix. The best scoring candidate mapping positions are stored for each read.

**Step 4 (mapping)**

The reads are mapped in the decreasing order of their score (most “trusted” reads first). When mapping a new read, the choice among the memorized candidate mapping positions depends on the reads that are already mapped to reference fragments which include these positions. Basically, the score and the interpretation of mismatches (reading errors, SNPs, indels) obtained for this read at *Step 3* are now recomputed according to a score-weighted multiple alignment of the mapped reads which it overlaps at each candidate position.

### 4.2. Comparison with Other Methods

In this section, we demonstrate the performance of our seeds on real data using our read mapping tool SToRM. We compare this performance with that of MAQ 0.7.1 [3], SHRiMP 1.3.2 [9] and 2.0, and PerM 0.2.6 [7] which are popular software tools for mapping SOLiD reads using the seed approach, and with two tools implementing the Burrows-Wheeler Transform approach: Bowtie 0.12.5 [11], and BWA 0.5.7 [12]. The tools were tested with their default settings, on a machine with 8 Intel Xeon CPUs running at 2GHz and 4G RAM.

Since the emphasis of this experiment is on seed accuracy, we run our implementation (SToRM) with the spaced seeds proposed by other seed-based tools in addition to the seeds designed with our method, in order to establish the quality of all the seed families in the same setup.

Table 1 shows seed families used in our experiments. SHRiMP-default is the default set of SHRiMP. PerM-F3-S20 is composed of seeds *F*_3_ and *S*_2,0_ of [7] taken together. Seeds 3-Lossy-12 and 3-Lossless-10-24p are two seed families of weights 12 and 10, respectively, designed with our method, using the following parameters for the underlying model: SNP probability 0.0085, indel probability 0.0015, reading error probability at the beginning of the read 0.01, reading error probability at the end of the read 0.1, periodic reading error probability 0.02. 3-Lossy-12 is the default seed family for SToRM, and 3-Lossless-10-24p are positioned seeds (24 positions) lossless for 1 indel and 1 SNP, 1 indel and 1 reading error, 2 SNPs, or 1 SNP and 2 reading errors.

#### 4.2.1. Read-Mapping Tools Comparison

Tables 2 and 3 show the results of experiments run, respectively, on 1,280,536 reads from *Saccharomyces cerevisiae* mapped on a 12,160,680 bp genome, and on 1,000,000 and reads from *Escherichia coli* mapped on a 4,573,347 bp genome (the latter is a public dataset available on the Applied Biosystems website).

 We can see that the approaches based on the Burrows-Wheeler Transform, Bowtie and BWA, are very fast but less accurate than most of the other tools. PerM is by far the fastest among the seed-based tools, thanks to its efficient periodic pattern indexing, but on the downside, it has a very poor sensitivity. This is caused by the fact that PerM seeds are designed lossless with respect to mismatches and SNPs but do not deal well with indels, most likely because of their large span. Note also that the quality of lossless seeds is mediocre on alignments exceeding their error threshold, as illustrated previously by Figure 12. Finally, SToRM has an advantageous percentage of mapped reads and execution time tradeoff among the seed-based tools.

#### 4.2.2. Seed Families Comparison

We further focused on the performance of the seed families described in Table 1, which we tested within our implementation on the aforementioned data sets. The test setup was identical for all seed families and consisted of the default SToRM parameters: the scores for match, mismatch, gap opening and gap extension were 5, −4, −6, −4, respectively, seeds were used with a single-hit strategy, the acceptance threshold score for the SIMD filter was 100, and obtained alignments were considered significant above the score 115. The results are given in Tables 4 and 5.

The PerM seeds have the smallest sensitivity, which is due, as mentioned above, to their large span and the fact that they are lossless for a small number of errors. These seeds would perform very well on data with few reading errors and no indels, but are unsuited for data with higher variations.

Comparing, within our program, the default seeds of SHRiMP of weight 12 (SHRiMP-default) with those with the same weight designed using our method, we observe a higher sensitivity of our seeds, reached with a smaller number of seeds, hence with less memory used for the index.

 Finally, the family of positioned seeds of weight 10 (3-lossless-10-24p) has a performance comparable to that of 3-lossy-12 in number of mapped reads, thus illustrating the advantageous sensitivity/selectivity tradeoff of positioned seeds depicted in Figure 11 and commented in Section 3.4. More precisely, we notice that the positioned seeds performed slightly better than 3-lossy-12 on the *S. cerevisiae* dataset, and slightly worse on the *E. coli* dataset, most likely because the periodic reading error bias is less obvious on the latter. Hence, we are confident that position-restricted seeds can be beneficial on data with a higher variation of reading error level, as well as for data sets with larger read lengths. Furthermore, while the execution time improvements brought by 3-lossless-10-24p in comparison with 3-lossless-12 are not impressive, the benefits are significant on the index size, since all the keys for a seed of weight 10 can be stored in 16 times less memory that the keys for a seed of weight 12.

## 5. Conclusions and Perspectives

In this paper, we presented a seed design framework for mapping SOLiD reads to a reference genomic sequence. Our contributions include the concept of position-restricted seeds, particularly suitable for short alignments with nonuniform error distribution; a model that captures the statistical characteristics of the SOLiD reads, used for the evaluation of lossy seeds; an efficient dynamic programming algorithm for verifying the lossless property of seeds; the ability to distinguish between SNPs, reading errors and indels in seed design.

 Our further work will include a more rigorous training of our models and in particular a more accurate estimation of involved probabilities, possibly using advanced methods of assessing the fit of a model. Another interesting question to study is the design of efficient combined lossy/lossless seeds which provide a guarantee to hit all the alignments with a specified number of errors and still have a good sensitivity when this threshold is exceeded. However, since lossless seeds tend to have a regular structure (see [19]) while best lossy seeds often have asymmetric and irregular structure, computing such seeds may be difficult.

## Figures and Tables

**Algorithm 1:**
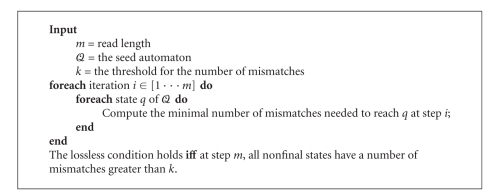
Lossless property verification algorithm.

**Table 1:** Seed families used in the experiments.

Seed set ID	Patterns	Positions
SHRiMP-default	#####- - -#######	All
	####- -###- -#- -####	
	###- -#- -#- - -###- -####	
	####- -#- - - -#- - -#- -#- -####	

PerM-F3-S20	###-#- -#- - -###-#- -#- - -##	All
	####- -#- - - -####- -#- - - -##	

3-Lossy-12	####-####-####	All
	####-###- -#- - - -####	
	####- - - -##- -##-####	

3-Lossless-10-24p	####-##-####	0,1,2,3,4,5,6,7,8,18,19,20
	#-########-#	2,12,15,16,18,19,20,21
	####-#- - - - - - -#-####	0,1,11,14

**Table 2:** Comparison of 6 read-mapping tools on *S. cerevisiae* dataset. The execution time refers to all steps, including index construction for the tools that can reuse the result of this step (Bowtie, BWA, MAQ).

Program	Mapped reads	Unique mapping positions	Execution time
Bowtie	553,140 (43.20%)	512,086 (39.99%)	0m50s
BWA	422,550 (33.00%)	395,342 (30.87%)	0m38s
MAQ	616,497 (48.14%)	567,549 (44.32%)	1m20s
PerM	418,524 (32.68%)	347,668 (27.15%)	0m31s
SHRiMP 1.3.2	663,923 (51.85%)	—	7m56s
SHRiMP 2.0	709,146 (55.38%)	—	1m22s
STORM	839,633 (65.57%)	754,402 (58.91%)	2m10s

**Table 3:** Comparison of 6 read-mapping tools on the *E. coli* dataset. The execution time refers to all steps, including index construction for the tools that can reuse the result of this step (Bowtie, BWA, MAQ).

Program	Mapped reads	Unique mapping positions	Execution time
Bowtie	456,416 (45.64%)	423,541 (42.35%)	0m18s
BWA	456,928 (45.69%)	424,239 (42.42%)	0m21s
MAQ	646,523 (64.65%)	588,362 (58.84%)	1m08s
PerM	413,102 (41.31%)	384,050 (38.40%)	0m23s
SHRiMP 1.3.2	687,855 (68.79%)	—	3m33s
SHRiMP 2.0	714,662 (71.47%)	—	0m41s
STORM	773,155 (77.32%)	697,164 (69.72%)	0m57s

**Table 4:** Comparison of 4 different seed families on the *S. cerevisiae* dataset.

Seed family	Mapped reads	Unique mapping positions	Execution time
PerM-F3-S20	768,732 (60.03%)	694,951 (54.27%)	0m55s
SHRiMP-default	836,899 (65.36%)	751,761 (58.71%)	2m15s
3-lossy-12	839,633 (65.57%)	754,402 (58.91%)	2m10s
3-lossless-10-24p	839,072 (65.53%)	755,208 (58.98%)	2m06s

**Table 5:** Comparison of 4 different seed families on the *E. coli* dataset.

Seed family	Mapped reads	Unique mapping positions	Execution time
PerM-F3-S20	731,447 (73.14%)	662,666 (66.27%)	0m33s
SHRiMP-default	772,861 (77.29%)	696,903 (69.69%)	1m02s
3-lossy-12	773,155 (77.32%)	697,164 (69.72%)	0m57s
3-lossless-10-24p	772,336 (77.23%)	696,615 (69.66%)	0m44s
